# Multi-omics analysis reveals the mechanism of enhanced GABA production in *Lactiplantibacillus plantarum* JY7 under acoustic and thermal coupling^☆^

**DOI:** 10.1016/j.ultsonch.2026.107969

**Published:** 2026-07-21

**Authors:** Wenying Yuan, Hao Zhang, Lixin Cai, Xiefei Li, Daodong Pan, Zhen Wu, Jie Luo, Maiquan Li, Xiankang Fan, Hui Zhou

**Affiliations:** a School of Public Health, The Key Laboratory of Environmental Pollution Monitoring and Disease Control, Ministry of Education, Guizhou Provincial Engineering Research Center of Ecological Food Innovation, Guizhou Medical University, Guiyang 561113, China; b College of Food Science and Technology, Hunan Agricultural University, Changsha 410128, China; c Key Laboratory of Animal Protein Food Processing Technology of Zhejiang Province, College of Food and Pharmaceutical Sciences, Ningbo University, Ningbo 315832, China; d College of Food Science and Nutritional Engineering, China Agricultural University, Beijing 100083, China; e Beijing Zhongke Chuangyuan Technology Co., Ltd, China

**Keywords:** Lactic acid bacteria, γ-Aminobutyric acid, Ultrasonic processing, Transcriptomics, Metabolomics

## Abstract

The efficient microbial biosynthesis of γ-aminobutyric acid (GABA) remains challenging. This study investigated the molecular mechanism for high GABA production by *Lactiplantibacillus plantarum* JY7 under acoustic-thermal coupling. *Lactiplantibacillus plantarum* JY7 (isolated from fish intestines) exhibited the highest GABA yield (1.0014 mg/mL) among 425 lactic acid bacteria strains, demonstrating excellent acid tolerance and bile salt tolerance activity. Under the optimized conditions (0.5 % L-MSG, 45 μmol/L PLP, 150 W, 38℃), the acoustic-thermal coupled field treatment increased GABA yield by 2.1-fold (0.5104 ± 0.0238 mg/mL) relative to the control. Acoustic-thermal coupling enhances cell membrane permeability, thereby boosting substrate uptake and product efflux. This process directly upregulates key GABA biosynthetic genes (*gadB/gadC*), activates energy metabolism to augment precursor and ATP supply, initiates cellular repair and defence to maintain homeostasis, and redirects carbon flux to GABA synthesis. This provides a theoretical basis for the development of GABA-rich fermented foods.

## Graphic abstract

**Figure:**
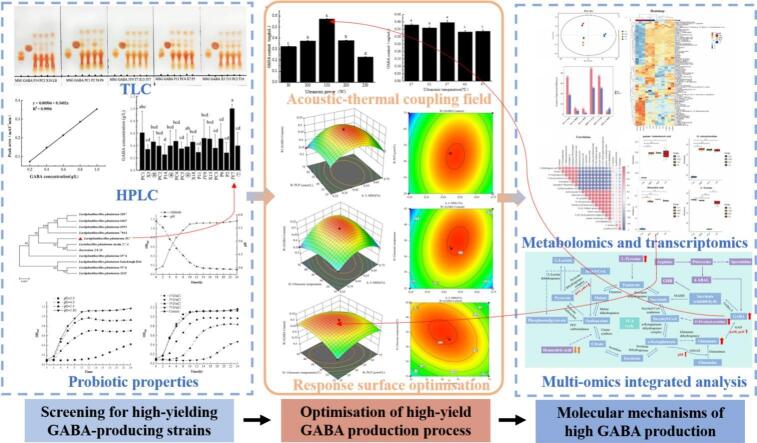

## 1 Introduction

Gamma-aminobutyric acid (GABA) is a pivotal inhibitory neurotransmitter essential for regulating emotional balance, stress response, and sleep-wake cycles [22]. Dysfunctions in the GABAergic system are closely associated with widespread health issues, such as anxiety and insomnia; for instance, sleep disorders are observed in approximately 50 % of anxiety patients, and sleep deprivation is known to exacerbate anxiety symptoms [10]. Oral GABA supplementation has been shown to elevate GABA levels in key brain regions, such as the prefrontal cortex, modulate anti‑inflammatory factors (e.g., IL-10, TGF-β1), enhance synaptic plasticity, and thereby alleviate anxiety‑like behaviors, suppress epileptic seizures, and promote neural repair [6], [14], [69]. The physiological roles of GABA are finely regulated and exhibit gender-specific effects, as evidenced by its promotion of growth hormone-releasing peptide expression in male mice [7]. Importantly, GABA modulates the gut microbiota in a strain-dependent manner, whereby beneficial genera such as *Lactobacillus* and *Bifidobacterium* are favoured whilst pathogens are suppressed. Consequently, intestinal barrier function and systemic metabolic homeostasis are strengthened via short-chain fatty acid regulation [12]. Natural GABA‑rich extracts (e.g., from *Curcuma zedoaria* [8] and fermented *Moringa oleifera* leaves [84] exert broad anti‑inflammatory actions through inhibition of the TLR-4/NF-κB pathway, which underscores the potential of natural GABA sources. Organ‑specific regulatory effects include the amelioration of liver inflammation via TLR4 suppression in Kupffer cells, and the exertion of antioxidant and anti-inflammatory activity in gastric tissue [80]. Through GABA_A_/GABA_B_ receptor activation and the gut-brain axis, neuronal excitability, immune responses, and metabolic regulation are balanced, highlighting the multidimensional health benefits of GABA and its high value in functional foods and pharmaceuticals [67].

Currently, industrial GABA production is achieved through three established methods: chemical synthesis, plant extraction and microbial fermentation [79]. Chemical synthesis involves harsh conditions and potential harmful residues, while plant extraction is costly due to low GABA content (0.03 %-0.1 %) in raw materials. By contrast, microbial fermentation, particularly the glutamate decarboxylase (GAD) system in lactic acid bacteria (LAB), converts L-glutamic acid into GABA under mild conditions, offering advantages of safety, clear metabolic pathways, and scalability [42]. Generally recognized as safe (GRAS) certification strains such as *Lactiplantibacillus plantarum* have become the mainstream strategy for food-grade GABA production [29]. However, wild-type strains typically exhibit low GABA yield (0.1–10 g/L) and substrate conversion rates below 50 %. Previous studies identified *Lactobacillus brevis* 54–1 as a GABA producer, and it was noted that GAD system activity is limited by environmental pH, substrate inhibition, and product feedback [18]. Although genetic engineering can be applied to enhance yield, engineered strains often suffer from genetic instability, which restricts industrial application [34]. Moreover, the regulatory networks involved in glutamic decarboxylase have not been fully elucidated.

To overcome these bottlenecks, acoustic-thermal coupling field technology has received widespread attention as an emerging physical fermentation enhancement strategy [83]. Shokri et al. [51] reported that low-frequency ultrasound treatment increases the growth of *Lactococcus lactis* subsp. *lactis* by 7.09 % to 23.22 %, and also increases β-galactosidase activity. This technology achieves precise regulation of microbial metabolism through synergistic physical stimulation of the thermal and acoustic field. The thermal component may influence cellular physiology by modulating membrane fluidity and potentially affecting the expression of stress‑responsive genes, including the *gad* gene cluster [28]. Low-frequency ultrasound generates mechanical effects including cavitation, micro-streaming, shear forces and acoustic streaming, which can transiently increase cell membrane permeability and enhance mass transfer [39], [46]. The synergistic effect of the thermal field and acoustic field may trigger global physiological responses in microorganisms by activating different signaling pathways (such as the heat shock factor pathway and the calcium signal pathway mediated by mechanosensitive ion channels), driving their metabolic network reprogram towards the GABA synthesis pathway [11]. However, the molecular mechanism through which this synergistic effect influences GABA metabolism in LAB at the transcriptional and metabolic levels remains unclear. Therefore, it is hypothesized that the acoustic-thermal coupling field may enhance GABA production in LAB by affecting the expression of GAD-associated genes.

This study investigated the molecular mechanisms governing GABA metabolism in *Lactiplantibacillus plantarum* under acoustic-thermal coupling conditions. First, high GABA-producing LAB were screened from raw materials including kimchi, yogurt and fish intestines, and the physiological and biochemical properties of the target strains were subsequently determined. Second, single-factor experiments and response surface methodology were employed to optimise conditions for efficient GABA enrichment by these strains under an acoustic-thermal coupling field. Finally, a multi-omics approach that combined transcriptomics and metabolomics was employed to elucidate the synergistic molecular mechanisms that regulate GABA metabolism. This work was conducted to decipher the regulatory network of GABA synthesis under combined physical stress, and providing a theoretical foundation for physical field-assisted fermentation was established [55].

## 2 Materials and methods

### 2.1 Experimental strains and equipment

The experimental strains were selected from samples including pickles, fish intestines, natural cheese, and kefir granules. Strain activation was carried out in MRS broth medium. The solid screening medium was prepared as MRS agar supplemented with 0.2 % bromocresol purple. The liquid screening medium was prepared by adding1 % (w/v) L-monosodium glutamate (L-MSG) to MRS medium. Ultrasound treatment was performed using a digital ultrasonic cleaner (PL-FS80T, Dongguan Konsung Ultrasonic Technology Co., Ltd., China), which operated at a fixed frequency of 40 kHz with a total nominal power of 500 W (adjustable from 1 % to 100 %). Temperature was controlled by the built-in heating system of the ultrasonic cleaner. The desired temperature was set on the digital control panel, and the water bath temperature was maintained within ± 1℃ of the set value by the heating system. The temperature of the bacterial suspension was verified before and during treatment with a digital thermometer.

### 2.2 Screening and characterization of high GABA-producing LAB

#### 2.2.1 Isolation and screening of GABA-producing LAB

Samples were homogenized with sterile normal saline, diluted in a gradient manner and spread onto MRS agar. The plates were incubated anaerobically at 37℃ for 36 to 48 h [68]. Colonies exhibiting a yellow halo (indicative of acid production) were selected, purified by streaking, and passaged three times to obtain pure cultures. For preliminary screening, strains were inoculated into liquid medium containing 1 % L-MSG and fermented at 37℃ for 60 h. The supernatant was collected and analyzed by thin-layer chromatography (TLC). According to the method of Sakkaa et al. [49] with modifications, the developing agent was prepared as n-butanol, acetic acid, and water at a ratio of 4:1:3 (v/v/v) containing 0.4 % ninhydrin. Plates were heated at 95℃ for color development, and GABA production was preliminarily assessed by comparing Rf values with standards.

#### Determination of GABA production and identification of high-yield strains

GABA production was quantified by high performance liquid chromatography (HPLC) [76]. Fermentation supernatants were derivatized with p-phenylenediamine and analyzed on a Phenomenex Luna C18 column. The mobile phase consisted of 20 mmol/L sodium acetate buffer (pH 7.3) and acetonitrile (4:1, v/v) at a flow rate of 0.8 mL/min, with detection at 334 nm. GABA concentration was calculated using a standard curve, and the highest-producing strain was selected for identification. Genomic DNA was extracted, and the 16S rRNA gene was amplified using primers 27F/1492R. The resulting sequence was subjected to NCBI BLAST analysis for species identification, and a phylogenetic tree was constructed with MEGA 11.

### 2.3 Physiological and probiotic characteristic analysis

Growth and acid production curves were determined as described previously [70]. Tolerance was assessed under the following conditions: acid stress (pH 3.0, 4.0, 5.0), osmotic stress (1 %-7 % NaCl, w/v), and bile salt stress (0.05 %-0.30 % bovine bile salts, w/v) for 6 h, with survival rate as the endpoint. Antibacterial activity against *Staphylococcus aureus* (ATCC 25923) and *Escherichia coli* (ATCC 25922) was evaluated by the agar diffusion assay. Survival in simulated gastrointestinal conditions was assessed using artificial gastric and intestinal fluids [59].

### 2.4 Effect of different factors on the GABA production

To explore the influence of key factors on GABA synthesis by LAB, a single-factor experiment was conducted first. The activated strains were inoculated at a 2 % (v/v) inoculation rate into MRS broth medium, and the effects of different levels of L-MSG concentration (0.25 %-3.0 %, w/v), cofactor pyridoxal 5′-phosphopyridoxal (PLP) concentration (25–200 μmol/L), and the processing parameters of acoustic-thermal coupling field including ultrasonic temperature (27-47℃), ultrasonic power (50–250 W), and ultrasonic treatment time (10–30 min) on GABA production were investigated. The factor levels were selected based on previous studies and preliminary experiments, covering typical optimal conditions while identifying inhibitory or damaging effects at higher levels[66]. For single-factor experiments, the baseline conditions were established as follows unless otherwise specified: L-MSG concentration 1.0 % (w/v), PLP concentration 50 μmol/L, ultrasonic temperature 37℃, ultrasonic power 150 W and ultrasonic time 20 min. Each factor was varied individually while the others were maintained at their baseline levels. Each group of experiments was performed in triplicate, and samples were taken after fermentation for GABA determination.

Based on single-factor results, four significant variables were selected: L-MSG concentration (A), PLP concentration (B), ultrasonic power (C) and ultrasonic temperature (D). The optimal values for these factors were further determined by a Box-Behnken design. A Box-Behnken design with 29 experimental runs (including 5 center points) was constructed with Design-Expert® 13.0 (Stat-Ease Inc., USA), with GABA yield as the response (Y). Model significance was evaluated by F-test and p-value, and factor interactions were visualized by three-dimensional response surface plots to determine optimal conditions.

### 2.5 Microscopic structure of the strain by scanning electron microscopy

The examination of cell morphology was performed as described by Fan et al. [19] with some modifications. Bacterial cells were fixed with 2.5 % glutaraldehyde at 4℃ overnight, washed three times with 0.9 % saline and dehydrated through a graded ethanol series (50 %, 70 %, 80 %, 90 %, 95 %, 100 %, 15 min per step). Samples were subsequently frozen at −80℃, vacuum freeze-dried for 12 h, sputter-coated with gold and observed under a scanning electron microscope (JSM-6380LV, JEOL, Japan).

### 2.6 Transcriptomics analysis

Samples for transcriptomic and metabolomic analysis were collected after 16–18 h of cultivation. Total RNA was extracted from the samples and 150 bp paired-end sequencing was conducted on the Illumina HiSeqTM platform. Raw reads were quality-filtered and adapter-trimmed with Trimmomatic. Clean reads were aligned to the reference genome of *Lactiplantibacillus plantarum* with Rockhopper2 [57]. Gene expression levels were calculated as FPKM values. Differentially expressed genes were screened with DESeq2 software (|log2FC| ≥ 1 and FDR ≤ 0.05), and functional enrichment analysis was performed through the KEGG and GO databases (corrected p-value ≤ 0.05). To validate the transcriptomic data, RT-qPCR was performed using the CFX Maestro system (Bio-Rad) with 16S rRNA as the internal reference. Relative expression levels of genes were expressed as log2FC relative to the Control group. All reactions were performed in triplicate. Primer sequences are listed in Table S1.

### 2.7 Metabolomics analysis

Non-targeted metabolomics analysis was conducted as described by Colas et al. [13]. Approximately 30 mg of the bacterial cells were taken and 400 μL of the methanol/water solution with internal standards (4:1) was added. The mixture was then ground with steel beads, extracted by ultrasonic treatment, and centrifuged. The supernatant was collected and separated on a UPLC-HSS T3 chromatographic column with elution by a gradient of 0.1 % formic acid in acetonitrile. Data were collected using the Q Exactive HF-X mass spectrometer in positive and negative ion modes. After peak extraction by XCMS, ions with a relative standard deviation > 30 % or a missing value rate > 50 % were excluded. The data were log2 transformed and subjected to principal component analysis (PCA), partial least squares discriminant analysis (PLS-DA), and orthogonal partial least squares discriminant analysis (OPLS-DA). Metabolites with variable importance in projection (VIP) > 1, fold change (FC) ≥ 1.2 or ≤ 0.833 and *p* < 0.05 were selected as differentially expressed metabolites. They were qualitatively identified using a self-built database (retention time deviation ± 0.3 min, fragmentation matching score ≥ 45), and KEGG pathway enrichment analysis was performed.

### 2.8 Statistical analysis

All experiments were conducted in triplicate (n = 3), and data were presented as mean ± standard deviation. Statistical analysis was performed with SPSS 26.0, and means were compared by one-way ANOVA followed by Tukey's HSD test at a significance level of p < 0.05. Response surface analysis was conducted with Design-Expert 13.0.

## 4 Results and discussions

### 3.1 Strain identification and performance characterization

#### 3.1.1 Strain screening and identification

**Fig. 1:**
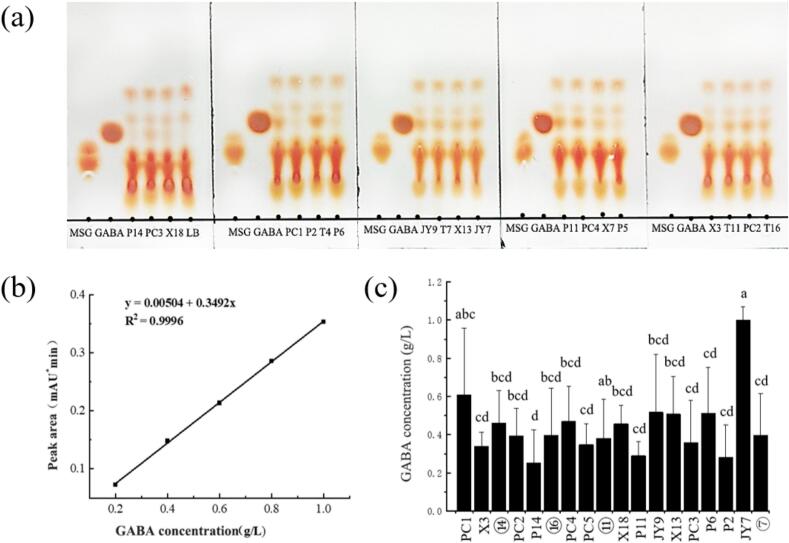
Screening and identification of a high GABA-producing LAB strain. (a) TLC of GABA-producing lactic acid bacteria isolates. (b) GABA standard curve for HPLC quantification. (c) GABA production by top-performing isolates cultivated in MRS broth supplemented with 1.0 % (w/v) L-MSG. Strain JY7 showed the highest yield (1.0014 ± 0.0724 g/L, equivalent to 1.0014 mg/mL).

**Fig. 2:**
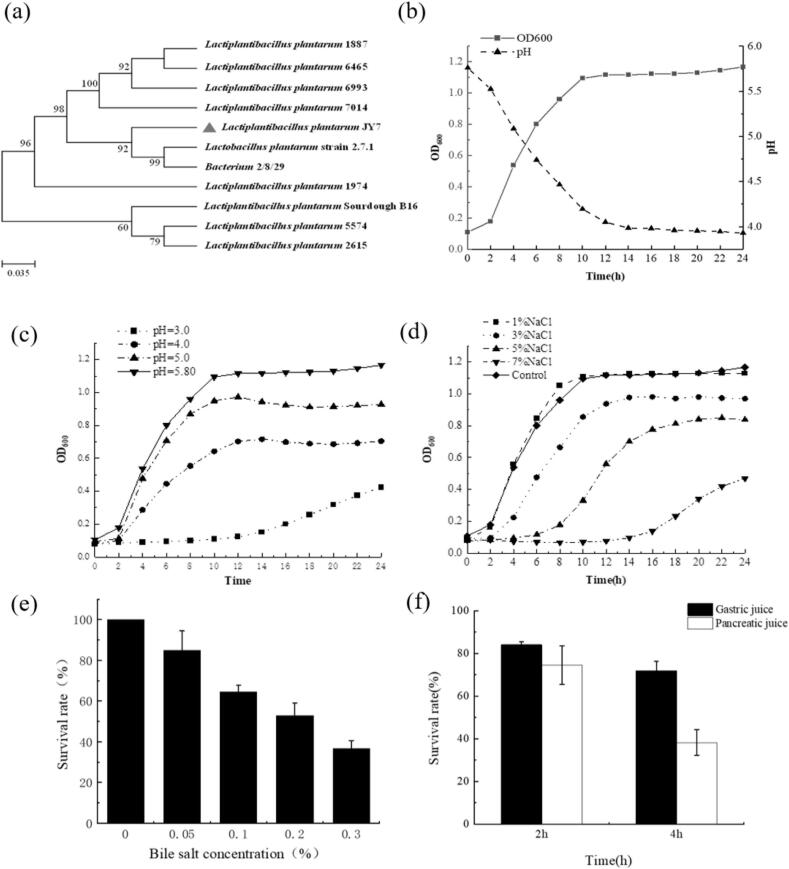
Phylogenetic and physiological characterization of *L. plantarum* JY7. (a) 16S rRNA-based phylogenetic tree. (b) Growth (OD_600_) and acidification (pH) profiles. (c) Acid, (d) osmotic, and (e) bile salt tolerance. (f) Simulated gastrointestinal survival. Data are mean ± SD (n = 3).

All 425 strains were subjected to preliminary screening by TLC. Representative TLC results from 20 of these strains that produced GABA clearly are shown in Fig. 1a. The remaining 405 strains either exhibited no detectable GABA or showed low GABA content. The concentration of GABA produced by the tested strains after 60 h of cultivation in MRS broth medium containing 1 % L-MSG was obtained from the GABA standard curve shown in Fig. 1b. Strains with significant GABA production from TLC screening were selected for quantitative screening by HPLC. The GABA production of the 18 strains with higher yields as determined by HPLC is shown in Fig. 1c. Among them, the yield of JY7 was particularly outstanding, with the GABA concentration reaching 1.0014 ± 0.0724 mg/mL. Compared with the *Lcb. rhamnosus* SN12 (0.82 mg/mL) reported by Yin et al. [73], JY7 demonstrated a significant advantage in GABA production. Therefore, strain JY7 was selected for subsequent experiments. The 16S rDNA sequence of strain JY7 was compared with the GenBank database, and the genes with high similarity were all identified as *Lactiplantibacillus plantarum*. A phylogenetic tree of strain JY7 was constructed, as shown in Fig. 2a. It was on the same branch as *Lactiplantibacillus plantarum* 2.7.1 and *Bacterium* 2/8/29, with a high degree of similarity.

#### 3.1.2 Physiological and probiotic property assessment

Fig. 2b presents the growth curve and acid production dynamics of *L. plantarum* JY7. A lag phase was observed from 0 to 2 h after inoculation. From 4 h onwards, the bacteria entered the logarithmic growth phase and proliferated significantly. After approximately 10 h of cultivation, the growth rate tended to level off and the stationary phase was reached. Corresponding to the growth process, JY7 exhibited strong acid production ability. At 8 h of cultivation, which corresponded to the end of logarithmic growth, the pH of the fermentation broth had dropped below 4.5. This indicates that the strain possesses high metabolic activity, which provides an important basis for its application in fermented foods [75].

As shown in Fig. 2c, the growth of *L. plantarum* JY7 was significantly inhibited under pH 3.0 conditions. However, normal growth was still observed at pH 4.0. The results in Fig. 2d indicate that when the osmotic pressure was lower than 5 % NaCl, strain growth was not significantly affected. When the concentration reached 7 %, JY7 was essentially unable to survive. Additionally, in the bile salt tolerance experiment (Fig. 2e), when the concentration of bovine bile salt was not higher than 0.2 %, the survival rate of JY7 remained above 55 %. When the concentration reached 0.3 %, the survival rate dropped to approximately 37 %. This indicates that the strain exhibits moderate bile salt tolerance and may have acceptable potential to remain active in the human gastrointestinal bile salt environment [61]. Fig. 2f further shows that when cultivated in simulated gastric and intestinal fluids, the survival rate of JY7 significantly decreased as the treatment time extended from 2 h to 4 h (*p* < 0.05). Nevertheless, the survival rate still remained above 40 % at 4 h, which indicates limited but acceptable tolerance to simulated gastrointestinal conditions [45]. These values are comparable to those of *L. plantarum* 54–1, a previously characterized probiotic strain[18]. The antibacterial activity determination showed that JY7 exerted good inhibitory effects on *Escherichia coli* and *Staphylococcus aureus*, with inhibition diameters of 17.36 ± 1.33 mm and 17.97 ± 1.59 mm, respectively. In conclusion, *L. plantarum* JY7 has strong acid production ability, can grow normally under pH 4.0 conditions, and shows good inhibitory effects on *Escherichia coli* and *Staphylococcus aureus*. Thus, the strain has the potential to serve as a candidate for subsequent research.

### 3.2 Effect of different factors on the GABA production by JY7

**Fig. 3:**
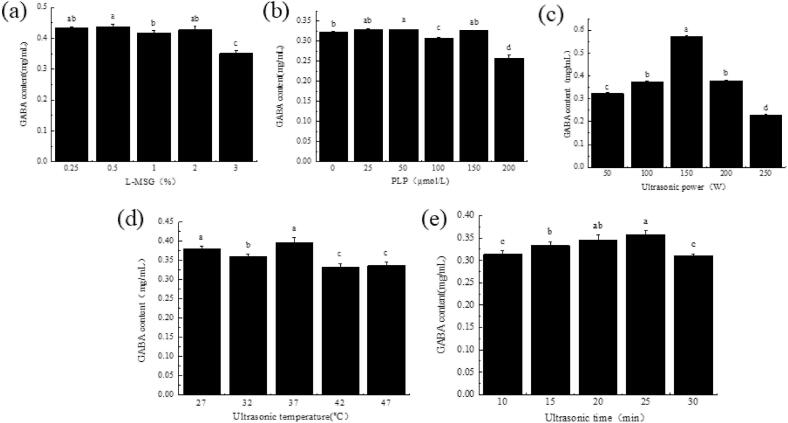
Single-factor optimization of GABA yield. Effect of (a) L-MSG (0.5–3.0 %, w/v), (b) PLP (25–200 μmol/L), (c) ultrasonic power (50–250 W), (d) ultrasonic temperature (27-47℃), and (e) ultrasonic time (10–30 min). Data are mean ± SD (n = 3).

Factors in this single-factor experiment were selected on the basis of the known or hypothesized roles of each factor in the GABA synthesis pathway of LAB and their responses to physical stress [53]. Specifically, these included the substrate L-MSG, the cofactor PLP, and the parameters of the acoustic-thermal combined field (ultrasonic temperature, power and time). L-MSG was employed as a direct precursor for GABA synthesis through the GAD pathway. The availability of extracellular L-glutamate is usually the main limiting factor for GABA production in microbial fermentation [20]. It has been consistently shown in various studies on GABA-producing LAB that the initial L-glutamate concentration is positively correlated with GABA production until the substrate reaches a high concentration and becomes inhibitory. In this study, GABA production was optimal when the initial L-MSG concentration was 0.5 %, and the production significantly decreased in a 3 % L-MSG medium (Fig. 3a). It was initially inferred that the high concentration of L-MSG caused a rapid increase in pH due to decarboxylation, which inhibited the growth and enzyme activity of LAB to some extent [62]. PLP is a necessary cofactor for GAD enzyme activity and acts as an electron sink during the decarboxylation reaction from L-MSG to GABA. The exogenous addition of PLP has been proven to increase the GAD activity of LAB, thereby increasing GABA production in strains including *Lactiplantibacillus plantarum* [16], [71]. As shown in Fig. 3b, the GABA production of *L. plantarum* JY7 showed significant differences when different concentrations of PLP were added. The highest yield was observed at a PLP concentration of 50 μmol/L, which reached 0.3278 ± 0.0012 mg/mL.

It is hypothesized that the application of the acoustic-thermal field can enhance GABA production through physicochemical effects on bacterial cells. Moderate temperature elevation during acoustic-thermal treatment may induce the expression of heat shock proteins and alter membrane fluidity. This is related to increased stress tolerance and potential upregulation of metabolic gene transcription, including those in the GAD system [48]. The selected range of 27-47℃ in this study included the optimal growth temperature of *Lactiplantibacillus plantarum*, while introducing controllable thermal coupling. The results showed that GABA production was optimal at 37℃, which is the generally optimal growth temperature of lactic acid bacteria. Low-frequency ultrasound acts mainly through the formation, growth, and implosion of microbubbles to exert its effect. This generates local extreme conditions (high temperature, high pressure, shear force), which reversibly increases the permeability of the cell membrane. This transient permeability is believed to promote the absorption of substrates (L-MSG and PLP) and the efflux of products (GABA), potentially reducing the feedback inhibition of the final product on the GAD system [40]. The power and duration of ultrasound treatment directly affect the degree of cavitation and its biological effects, and these parameters require optimization. Therefore, the effects of ultrasonic power (50–250 W), ultrasonic temperature (27-47℃) and ultrasonic time (10–30 min) on the production of GABA by *L. plantarum* JY7 were investigated (Fig. 3c, 3d, 3e), it was found that the optimal conditions were 150 W, 37℃, and 25 min of ultrasonic treatment. These single-factor results informed the selection of factors and the definition of experimental levels for the subsequent Box-Behnken design, through which the true optimal conditions were determined.

### 3.3 Response surface analysis on the GABA production by JY7

#### 3.3.1 Response surface design

Based on the single-factor experiments, L-MSG (A), PLP (B), ultrasonic power (C), and ultrasonic temperature (D) were selected for response surface optimization experiments. Ultrasonic treatment time, although significant in single-factor tests, exhibited a relatively narrow optimal range and a smaller effect magnitude. Therefore, it was fixed at its optimal value of 25 min in the subsequent Box-Behnken design. The response surface method is a set of mathematical and statistical techniques that is used to establish empirical models [5]. A fermentation time of 24 h was used for all response surface and verification experiments, as preliminary time-course experiments indicated that GABA accumulation peaked at 24–30 h, beyond which GABA degradation and reduced cell viability were observed. The experimental design and results are shown in Tables S2 and S3. A quadratic polynomial regression equation was obtained by multiple regression fitting with Design-Expert software:

$$Y=0.5023+0.0186*A-0.0072*B+0.0037*C+0.0127*D+0.0162*AB-0.0067*AC+0.0261*AD-0.0146*BC-0.0252*BD-0.0027*CD-0.0767*A^{2}-0.0358*B^{2}-0.0379*C^{2}-0.0415*D^{2}$$

where A, B, C and D are the independent variables for L-MSG, PLP, ultrasonic power, and ultrasonic temperature, respectively.

The variance analysis results of the regression equation were obtained through regression analysis of the response function (GABA production) combined with variable tests (Table S4). In the model, a smaller P value and a larger F value indicate higher significance. The F value of the GABA production model was 143.94 (*p* < 0.0001), which indicates that the selected quadratic polynomial model was highly significant and that the response surface method is reliable. The P value of the lack-of-fit term was 0.8334 (> 0.05), indicating that the lack of fit was not significant. Unknown factors had little interference with the experimental fitting, and the experimental error was mainly attributed to random errors. The model showed excellent goodness of fit, with R^2^ = 0.9931, adjusted R^2^ = 0.9862 and predicted R^2^ = 0.9732. The close agreement between adjusted and predicted R^2^ (difference = 0.013) indicated no overfitting. The low coefficient of variation (1.30 %) and high adequate precision (38.97) further confirmed the reliability and predictive capability of the model, which is consistent with previous response surface studies that also reported high R^2^ values [65]. The optimal values of each experimental variable are as follows: L-MSG concentration 0.5357 %, PLP concentration 45.821 μmol/L, ultrasonic temperature 38.259℃ and ultrasonic power 152.938 W. Under these conditions, the GABA content was predicted to be 0.5059 mg/mL. Based on practical operation, the L-MSG concentration was selected as 0.5 %, PLP concentration as 45 μmol/L, ultrasonic power as 150 W and ultrasonic temperature as 38℃ for the verification experiment. Under these conditions, the GABA content was 0.5104 ± 0.0238 mg/mL (mean ± standard deviation; n = 3), and the validity of the model was confirmed. Accordingly, this response surface-optimized condition was used in all subsequent experiments.

#### 3.3.2 Response surface interaction analysis

**Fig. 4:**
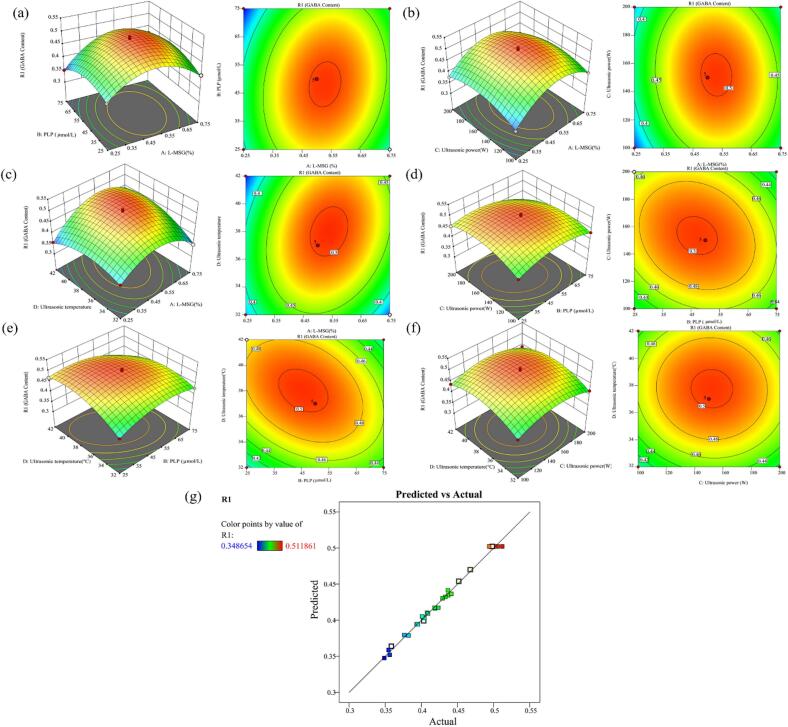
Response surface analysis of interactive effects on GABA yield. Surface and contour plots illustrate significant interactions between (a) L-MSG and PLP, (b) L-MSG and ultrasonic power, (c) L-MSG and ultrasonic temperature, (d) PLP and ultrasonic power, (e) PLP and ultrasonic temperature, and (f) ultrasonic power and ultrasonic temperature. (g) Predicted vs. actual plot. All factors were coded to dimensionless values (−1, 0, +1) corresponding to the ranges specified above. The model was validated with 5 center-point replicates (n = 3 per run). Statistical significance was determined by ANOVA (*p* < 0.05).

This study utilized Design-Expert 13.0 software to construct a mathematical model between the independent variables and the dependent variable. The influence of each variable and their interactions on GABA content synthesized by *Lactiplantibacillus plantarum* was systematically presented through response surface plots and contour maps. As shown in Fig. 4a-4f, the response surface exhibited a clearly convex shape with a steep and downward-opening surface, which indicates that a definite maximum GABA yield exists within the four factors examined in the experiment. Meanwhile, the contour lines were distributed in an elliptical pattern, which confirmed that GABA yield was significantly affected by the interactions between L-MSG and PLP, ultrasonic power and L-MSG, ultrasonic temperature and L-MSG, and PLP and ultrasonic power. As shown in Fig. 4g, a good alignment between experimental and predicted GABA yields was observed, which confirmed the reliability of the model. This validated quadratic polynomial model is therefore suitable for predicting and optimizing GABA production.

In the response surface, the effects of each factor were intertwined, and their influence levels could be intuitively reflected through the density of the contour lines and the slope of the response surface [32]. Specifically, denser contour lines and a steeper response surface indicated greater influence of that factor or interaction, whilst wider spacing of the contour lines indicated smaller influence. Further analysis of the interactions revealed that when the temperature was at a low level (32℃), an increase in L-MSG concentration did not significantly enhance GABA yield. However, when the temperature rose to a high level (42℃), increasing the L-MSG concentration significantly promoted GABA synthesis. This indicates that an appropriate temperature may enhance the affinity or catalytic efficiency of glutamate decarboxylase for the substrate, which reflects the crucial role of temperature in regulating the kinetics of enzymatic reactions [2]. Similarly, under low-temperature conditions, increasing PLP concentration had a positive impact on yield, whilst under high-temperature conditions, excessive PLP inhibited synthesis. This suggests that temperature may affect the binding stability or spatial conformation of PLP and enzyme protein [58]. Moreover, under fixed ultrasonic parameters, simultaneously increasing L-MSG and PLP did not exhibit a simple additive effect. This suggests that competitive binding or metabolic flux distribution phenomena may exist between the substrate and coenzyme, and that the inhibitory effect caused by excessive single component should be avoided [41]. These results collectively indicate that the mechanical effects generated by ultrasonication, such as cavitation and shear, may regulate GABA biosynthesis by influencing the intracellular transfer efficiency of PLP or the active conformation of the enzyme-coenzyme complex. Process optimisation should therefore focus on seeking the optimal balance range of multi-factor synergy rather than pursuing extreme conditions of a single factor.

### 3.4 SEM

**Fig. 5:**
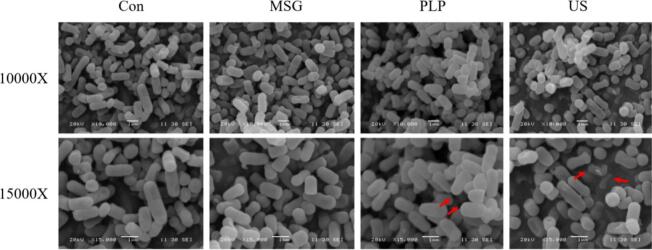
SEM images of *L. plantarum* JY7 cells: Control group (Con), L-MSG supplemented group (MSG), PLP supplemented group (PLP), and thermosonication group (US). Con: basal MRS; MSG: +0.5 % L-MSG; PLP: +0.5 % L-MSG + 45 μmol/L PLP; US: +0.5 % L-MSG + 45 μmol/L PLP, ultrasound (150 W, 25 min, 38℃).

To evaluate the cumulative effects of substrate supplementation, cofactor addition and acoustic-thermal treatment on cell morphology, four experimental groups were designed in a stepwise manner: Con (basal MRS medium), MSG (MRS + L-MSG), PLP (MRS + L-MSG + PLP) and US (MRS + L-MSG + PLP + acoustic-thermal treatment). The morphology of *L. plantarum* JY7 under four treatment conditions was observed by scanning electron microscopy (Fig. 5). The results showed that different treatments had significant effects on bacterial morphology, abundance and integrity. In the control group (Con), the cells presented a typical short rod shape and were mainly dispersed, which was consistent with the typical morphology of *L. plantarum* when cultured in standard MRS broth [35]. However, the cell surfaces of some bacteria in the control group were slightly rough, which might be attributed to the nutrients of the culture medium [60]. In contrast, significant promotion of bacterial growth was observed in both the MSG group and the PLP group. The addition of monosodium glutamate, which serves as a key amino acid and nitrogen source, significantly increased cell density and rendered the cell morphology more plump and uniform [31]. This is consistent with studies that suggest glutamate and its salts enhance the growth and metabolic activity of LAB by improving nitrogen absorption and energy metabolism. The further addition of PLP, which is the active coenzyme form of vitamin B6, also appeared to enhance this effect. PLP is a necessary cofactor for many enzymes involved in amino acid metabolism, and its supplementation has been reported to optimize cell metabolism and promote vigorous microbial growth [33]. Additionally, the cells in the MSG group and the PLP group showed a tendency to aggregate, and clusters or micro-colonies of varying sizes were formed. This aggregation phenomenon indicates that the nutrient-rich environment not only stimulates cell proliferation but may also induce early biofilm formation or cell adhesion. Nutrient-rich conditions have been proven to promote the production of extracellular polymeric substances by many bacterial species, including lactic acid bacteria, and to facilitate cell aggregation [47]. Therefore, the SEM results indicate that adding L-MSG and PLP optimised the metabolic environment of *L. plantarum* JY7, promoted its proliferation and cell aggregation, and this may be related to the enhancement of biofilm formation ability.

The most significant morphological changes were observed in the US group, which was treated with the combined ultrasonic and thermal fields. Compared to the PLP group, some *L. plantarum* JY7 cells in the US group exhibited altered surface morphology, including roughening and surface indentation [24]. This is consistent with previous reports on ultrasound-treated lactic acid bacteria [17]. Similarly, Shokri et al. [51] showed that the percentage of intact cells of *Lactococcus lactis* subsp. *lactis* after ultrasonic treatment decreased by 3.03–22.88 %. Ultrasonic irradiation generates complex physical fields, including high pressure, heat, micro-streams, and micro-vortices, which are collectively known as acoustic cavitation [82]. These mechanical effects can transiently affect the integrity of the bacterial cell envelope, which leads to increased membrane permeability without necessarily causing cell death [56]. Importantly, the US group maintained a viable cell count of 6.74 × 10^8^ CFU/mL, with a survival rate of approximately 86.38 %. This indicated that a substantial proportion of cells remained metabolically active after treatment. It is proposed that the morphological changes observed by SEM reflect transient permeabilization of the cell envelope, which enhances substrate (L-MSG) uptake and product (GABA) efflux, rather than irreversible cell damage.

### 3.5 Transcriptomic analysis

**Fig. 6:**
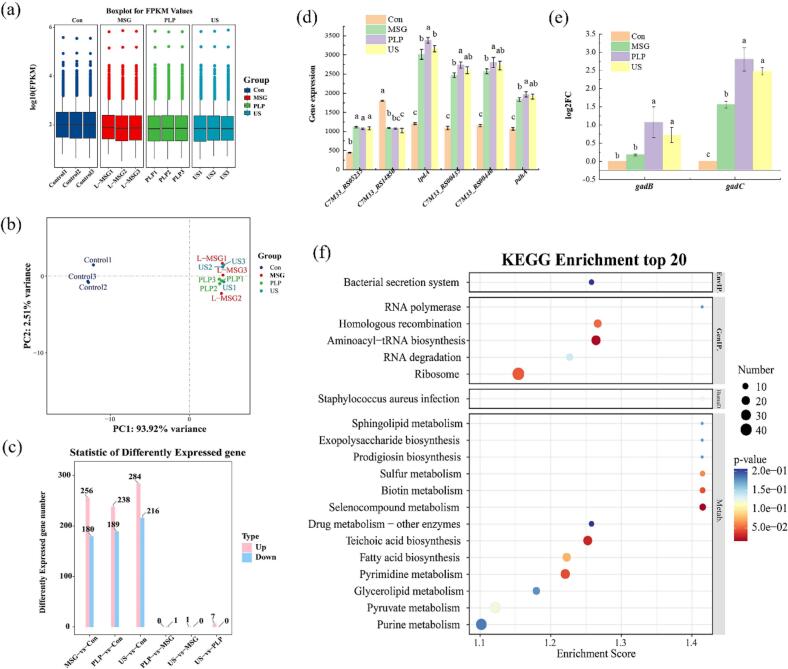
Transcriptomic analysis. (a) Gene expression distribution. (b) PCA score plot. (c) Differentially expressed gene (DEG) counts. (d) The bar chart of key DEGs. (e) RT-qPCR validation of *gadB* and *gadC*. (f) KEGG pathway enrichment. Groups and conditions as in Fig. 5.

The quality of transcriptomic data was assessed, and the gene expression abundance distribution across all samples was found to exhibit a typical unimodal pattern (Fig. 6a). This indicates that the data quality was reliable and suitable for subsequent analysis [36]. The read mapping statistics for each sample against the *Lactiplantibacillus plantarum* reference genome are summarized in Supplementary Table S5. In the principal component analysis, the first two principal components together explained over 96 % of the transcriptomic variation (Fig. 6b). Samples from the Con group clustered tightly, which demonstrates good biological reproducibility. The MSG group was positioned close to the Con group, which suggests that the addition of L-MSG alone induced a relatively mild transcriptional reprogramming. In contrast, the PLP group showed clear separation from both the Con and MSG groups along the PC1 axis. The US group exhibited a distinct distribution across both PC1 and PC2 dimensions, which indicates that both PLP and ultrasonic treatments triggered substantial transcriptional reprogramming. The biological significance of these spatial patterns was validated by the results of differentially expressed gene (DEG) analysis (Fig. 6c). The highest number of DEGs was identified in the comparison between the US and Con groups, and this number was significantly greater than that identified in the comparison between the MSG and Con groups. This statistically confirms that the US treatment was the core stage that activated a global transcriptional response [50].

In-depth analysis of key genes further elucidated the molecular details of this network (Fig. 6d). Significant up-regulation of genes encoding core components of the pyruvate dehydrogenase complex (e.g., *lpdA, pdhA*) indicated enhanced conversion of the glycolytic product pyruvate to acetyl-CoA. This thereby channeled more carbon flux into the TCA cycle and energy generation [81]. The pyruvate dehydrogenase complex is a critical gateway connecting glycolysis to the tricarboxylic acid (TCA) cycle. Its up-regulated activity directly suggests that the cell redirects more carbon flux from the end of glycolysis towards the TCA cycle and oxidative phosphorylation pathways, which generate energy and reducing power. Similarly, Yao et al. [72] reported that when microorganisms face high demand for precursor and energy consumption in anabolism, they remodel carbon flux distribution by up-regulating key node genes in central carbon metabolism to ensure sufficient supply of energy and carbon skeletons. To validate the transcriptomic data, we performed RT-qPCR analysis of the key GABA synthesis genes *gadB* and *gadC* under different treatment conditions. The RNA‑seq data showed that *C7M33_RS00435* and *C7M33_RS00440* were significantly up‑regulated in the PLP group and US group. Functionally annotated as glutamate decarboxylase and glutamate/GABA antiporter, these two genes matched the respective functions of *gadB* and *gadC.* Based on these annotations, we designed RT‑qPCR primers targeting these two loci for validation. The results showed that the relative expression patterns obtained by RT‑qPCR were generally consistent with the RNA‑seq data (Fig. 6e), with both methods confirming the transcriptional up‑regulation of *gadB/gadC* homologs in response to PLP supplementation. Notably, the transcript levels of these *gadB/gadC* homologs in the PLP group were consistently higher than those in the US group in both RNA‑seq and RT‑qPCR datasets, indicating that while acoustic‑thermal treatment further enhanced GABA yield at the product level, the transcriptional activation of the GAD system was most pronounced upon PLP cofactor supplementation. Beyond the GAD system itself, genes involved in glutamate supply were also differentially regulated. The gene encoding the large subunit of glutamate synthase (*C7M33_RS05235*) was up-regulated. This enzyme is responsible for the *de novo* synthesis of glutamate, which is the direct precursor of GABA. Glutamate is a direct precursor for GABA synthesis, and its intracellular concentration is one of the rate-limiting factors. The GS-GOGAT cycle, formed by glutamate synthase and glutamine synthetase, is the primary pathway for microbial assimilation of inorganic nitrogen and glutamate synthesis [63]. The up-regulation of this gene implies that the cell strengthened its *de novo* glutamate synthesis capacity from the source of nitrogen assimilation and carbon skeleton integration. This thereby provides ample substrate for the downstream GAD enzyme and avoids a synthesis bottleneck due to precursor shortage. Furthermore, the expression of a gene predicted to encode an FAD-dependent oxidoreductase (*C7M33_RS14850*) was changed, which suggests adjustments in intracellular redox balance. Although GABA synthesis does not directly consume reducing power, the maintenance of a high rate of amino acid synthesis, nitrogen assimilation, and the cell's response to potential stress requires a stable pool of NAD(P)H/FADH2[78]. This oxidoreductase might be involved in cofactor regeneration or related metabolic pathways, and its expression change might help maintain the redox homeostasis required for anabolism. Transcriptomic analysis also revealed that classical heat shock protein genes were activated by the acoustic-thermal coupling field. Specifically, *groEL* and *dnaK* were significantly upregulated in the US group compared with the Con group, with log_2_FC values of 0.9170 (adjusted p-value = 4.38 × 10^-76^) and 0.3715 (adjusted p-value = 3.51 × 10^-11^), respectively. The coordinated upregulation of *groEL* and *dnaK* provides convergent evidence that the acoustic-thermal field imposes detectable proteostatic stress on the cells. This stress likely arises from combined physical factors, including mild thermal exposure at 38℃ and localized shear forces from ultrasonic cavitation, both of which can induce protein misfolding or aggregation. Therefore, the upregulation of these chaperone genes indicates cellular stress perception, although this response cannot be attributed solely to thermal stress, as the combined physical perturbations, including mild thermal exposure at 38℃ and localized shear forces from ultrasonic cavitation, likely contribute collectively to the observed proteostatic stress response.

KEGG pathway enrichment analysis based on all differentially expressed genes (Fig. 6f) integrated the aforementioned findings from a systems perspective. This revealed the multidimensional and coordinated transcriptional reprogramming network that was triggered by the acoustic-thermal field treatment. The significant enrichment of pyruvate metabolism and purine and pyrimidine metabolism pathways directly echoed the up-regulation of pyruvate dehydrogenase complex genes (*lpdA, pdhA*) mentioned earlier. This marks the remodeling of central carbon metabolic flux to maximize the generation of acetyl-CoA from glycolytic products, thereby injecting more carbon flux into the TCA cycle to ensure sustained high ATP production [23]. Simultaneously, purines and pyrimidines are key components of ATP, GTP and nucleic acids. The activation of their metabolism not only meets the high energy consumption demand but also provides raw materials for rapid transcription and replication, supporting the cell’s overall high metabolic state. The enrichment of ribosome and aminoacyl-tRNA biosynthesis pathways is a classical marker of enhanced cellular translational capacity [37]. Efficient GABA synthesis requires the high expression of GAD and its associated transport and metabolic enzyme systems. The activation of these pathways indicates that substantial resources are invested by the cell in the synthesis of new protein machinery to meet the urgent need for rapidly converting transcripts into functional proteins, which is the material basis for achieving high-rate catalytic conversion. Furthermore, the dynamic remodeling of cell structure reflects an active adaptation to physical field stimuli [77]. The activation of pathways such as fatty acid biosynthesis, glycerolipid metabolism and teichoic acid biosynthesis strongly suggests that the cell is adjusting its membrane lipid composition and cell wall structure [3]. The shear forces and cavitation effects generated by the acoustic-thermal field might cause physical disturbance to the cell envelope. The up-regulation of these pathways aids in damage repair, adjusts membrane fluidity and potentially alters membrane permeability. Additionally, the enrichment of cofactor synthesis pathways such as sulfur metabolism and biotin metabolism plays a crucial functional role. Sulfur is an essential element for various cofactors, including iron-sulfur clusters and amino acids, and biotin is a key coenzyme for carboxylation reactions [52]. The activity of numerous metabolic enzymes, including GAD, is highly dependent on specific cofactors. This implies that the cell reinforces *de novo* cofactor synthesis to maintain the entire metabolic enzyme network, especially the core GABA synthesis enzyme system, in an optimal catalytic state. This thereby prevents cofactor shortage from becoming a new rate-limiting step.

It should be noted that transcriptional up‑regulation of *gadB/gadC* does not necessarily translate directly to proportional increases in functional GAD enzyme activity. It is plausible that the end‑product GABA may exert competitive feedback inhibition on GAD, and the substantial GABA accumulation in the PLP and US groups might partially counteract the PLP stimulatory effect, potentially explaining the non‑linear relationship between transcript abundance and final GABA yield. Additionally, post‑translational modifications or protein degradation rates might modulate GAD enzyme stability and catalytic efficiency, warranting future proteomic and enzymatic assays for further elucidation.

### 3.6 Metabolomics analysis

**Fig. 7:**
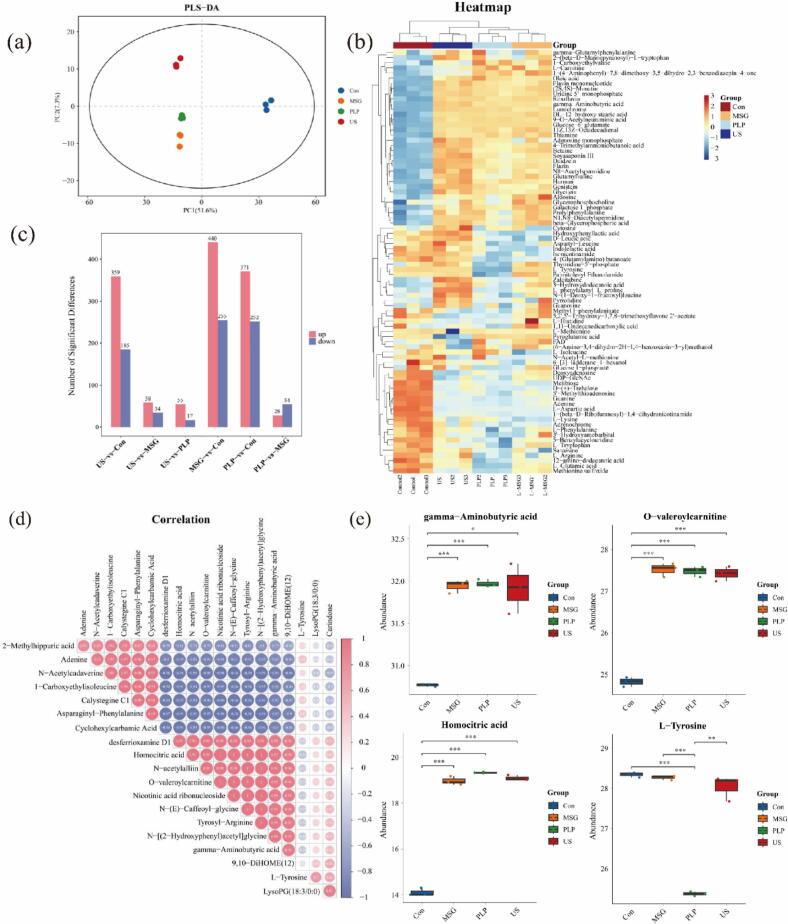
Untargeted metabolomics. (a) PCA score plot. (b) Heatmap of differential metabolites. (c) Differential metabolite counts. (d) Top 20 metabolite correlation network. (e) Box plots of key metabolites. Groups and conditions as in Fig. 5.

To clarify the metabolic mechanism by which the acoustic-thermal coupling field enhances GABA synthesis in *L. plantarum* JY7, untargeted metabolomics was employed. PCA score plots were generated (Fig. 7a), samples within the same treatment group were tightly clustered, which indicated high reproducibility and reliability of the experiment. Samples from different treatment groups were clearly separated along the principal component axes, which showed that successive interventions induced significant global metabolic reprogramming in the cells [43]. Heatmap analysis clearly distinguished the metabolic profiles of the four sample groups. Differential metabolites between groups were identified on the basis of the criteria of VIP > 1, *p* < 0.05, and FC ≥ 1.5 or ≤ 0.667 (Fig. 7b). In the comparison between the MSG group and the control group (Con), 695 differential metabolites were found (440 up-regulated, 255 down-regulated, Fig. 7c). This metabolic change was mainly attributed to the addition of L-MSG to the medium, which served as the direct substrate for GABA synthesis. This addition initiated early metabolic adaptation and network rearrangement [21]. KEGG pathway enrichment analysis was performed, and the differential metabolites were significantly enriched in pathways related to energy metabolism (e.g., glycolysis/gluconeogenesis), membrane phospholipid metabolism (e.g., glycerophospholipid metabolism), and polyamine biosynthesis. This suggested that substrate supplementation not only provided precursors but also activated basal energy production and the synthesis of cell membrane structural components [26]. Importantly, in the comparison between the US group and the Con group, GABA itself was identified as a key differential metabolite and was significantly elevated. This provided the most direct molecular evidence for the GABA accumulation observed. This process was accompanied by the elevation of its direct precursor L-glutamate, along with amino acids such as L-tyrosine and nucleotide precursors such as adenine, among others. These findings collectively indicated the comprehensive activation of the core biosynthetic pathway [74]. Concurrently, metabolites related to oxidative stress, such as N-acetylmethionine sulfoxide, were detected. This suggested that the acoustic-thermal coupling treatment might also have induced mild oxidative stress whilst driving synthesis, which triggered a cellular stress response [27]. A further comparative analysis was conducted between the US group and the PLP group. In the differential metabolite profiles, significant enrichment of plant-derived secondary metabolites was observed. These included flavonoids such as apigenin derivatives, phenylpropanoids such as coniferin, and lipid signalling molecules such as lysophosphatidylcholine and sphingosine. These metabolic alterations were attributed to reinforcement of a protective cellular environment that consolidated the previously established high-capacity metabolic state. The accumulation of flavonoids and phenylpropanoids was linked to activation of endogenous antioxidant defence systems that presumably mitigated reactive oxygen species generated by cavitation [4], [30]. The enrichment of sphingolipid-related metabolites and lysophosphatidylcholine was indicative of membrane lipid remodelling and mechanosensitive signalling, which was consistent with the transcriptional evidence. These protective adaptations were considered to sustain the high GABA flux that had been established by PLP and thereby to enhance and consolidate the efficiency of GABA synthesis. Notably, although the core GABA biosynthetic pathway had been fully activated by PLP, the GABA yield in the US group was further elevated compared with the PLP group. This demonstrated that the acoustic-thermal coupling field exerted a positive effect on GABA production enhancement beyond the cofactor-driven stage.

Based on the key metabolite correlation analysis of the four sample groups, an interaction network among the top 20 metabolites was constructed (Fig. 7d). This visually revealed the synergistic and antagonistic relationships in metabolite regulation across different treatment stages. Box plot analysis confirmed that the metabolic reprogramming was distinctly stage-specific (Fig. 7e). GABA and its associated precursor L-tyrosine were found to be significantly accumulated in the US treatment group. This verified that the acoustic-thermal coupling field was the key physical driver triggering the final synthesis burst. Notably, L-tyrosine is not only a precursor for protein synthesis, but its metabolic shunting can also provide carbon skeletons for aromatic amino acid-derived pathways. Its synchronous accumulation with GABA suggested that the cell might coordinately regulate multiple amino acid metabolic networks to fully support GABA synthesis. This observation is consistent with the global reprogramming of amino acid metabolism observed in microbial metabolic engineering to support target product synthesis [15]. The energy metabolism marker O-pivaloylcarnitine was maintained at a relatively high level after MSG treatment, which indicated that precursor addition initiated an energy preparation state that provided sustained support for subsequent energy-consuming reactions. Carnitine and its derivatives are primarily responsible for fatty acid transport and β-oxidation within cells, and they serve as key indicators of energy metabolism activity [25]. The early accumulation of this metabolite in the MSG group showed that the addition of the precursor L-MSG not only provided substrate but also rapidly initiated a cellular energy preparation state. This offered continuous energy support for the subsequent high energy-consuming biosynthetic reactions (e.g., GABA active transport, stress response) in the PLP and US stages. The changes in homocitrate revealed a dynamic redistribution of central carbon metabolism. Its accumulation in the MSG stage reflected an overall enhancement of carbon flux due to substrate addition. Its relative decrease in the PLP stage indicated that a large portion of carbon skeletons was diverted to specific pathways such as GABA synthesis. The restoration of its level to a balanced state in the US stage suggested that after intense adjustment, the metabolic network reached a new homeostatic balance that supported efficient synthesis. Homocitrate is an intermediate in the lysine biosynthesis pathway, and its fluctuations directly reflect the dynamic reallocation of resources by central carbon metabolism to meet synthetic demands. This is an adaptive metabolic strategy employed by microorganisms in response to environmental and metabolic pressures [38]. These data delineate a clear metabolic trajectory from metabolic preparation and core synthesis to system optimisation.

### 3.7 Multi-omics joint analysis of the synergistic enhancement mechanism

**Fig. 8:**
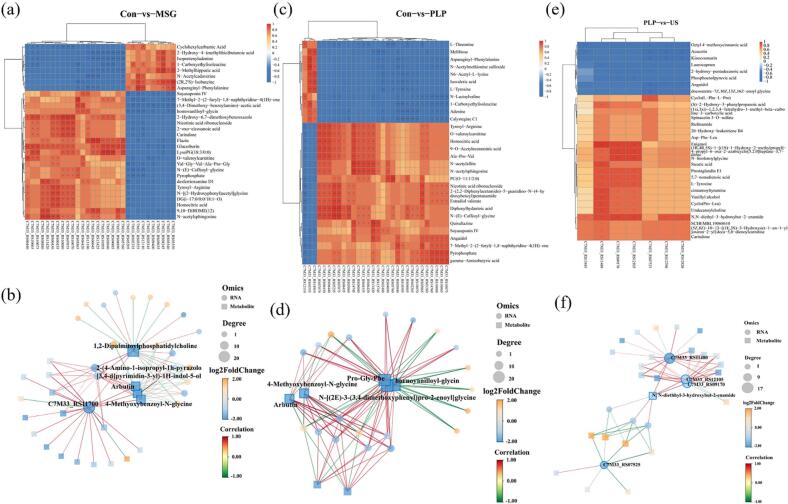
Integrated multi-omics correlation networks for the three treatment stages. (a,b) Con vs MSG, (c,d) Con vs PLP, (e,f) PLP vs US. Groups and conditions as in Fig. 5.

To further elucidate the synergistic mechanism by which the acoustic‑thermal coupling field promotes GABA synthesis in *L. plantarum* JY7, an integrated analysis of transcriptomic and untargeted metabolomic data was performed. This approach moves beyond the limitations of single‑omics descriptions, systematically revealing the progressive characteristics and internal logic of metabolic reprogramming at different stages from the perspective of dynamic gene-metabolite interaction networks. Correlation network analysis showed that during the MSG treatment stage, genes related to fatty acid metabolism, membrane phospholipid metabolism and polyamine metabolism formed significant early co-variation modules with their corresponding metabolites (Fig. 8a and 8b). These genes included those encoding phospholipid synthases and acetyltransferases, and the metabolites included lysophosphatidylglycerol (LysoPG) and O-pivaloylcarnitine. The accumulation of polyamines such as spermidine is an early stress marker in bacteria responding to environmental changes, and is closely linked to membrane stability [44]. This preparatory phase established the necessary membrane structural foundation and potential for sustained energy supply to support subsequent high‑intensity synthesis.

In the critical PLP treatment stage, the integrated analysis clearly revealed a highly coordinated core synthesis module (Fig. 8c, d). The central feature of this module was the absolute dominance of the glutamate metabolic pathway, which was manifested by strong positive correlations between key metabolites (GABA and glutamate) and core genes (*gadB*, *gadC*) of the GAD system [9]. This directly confirms the specific drive and enhancement of the GABA synthesis pathway by PLP as a cofactor. At the same time, the up‑regulation of key genes (*pdhA, lpdA*) that channel pyruvate into the TCA cycle was highly correlated with changes in energy carriers (O‑pivaloylcarnitine) and TCA-cycle intermediates (homocitrate). The co‑variation between purine synthesis‑related genes and ATP/adenine precursors provided immediate and sufficient ATP supply for the energy-intensive decarboxylation reaction catalysed by GAD.

**Fig. 9:**
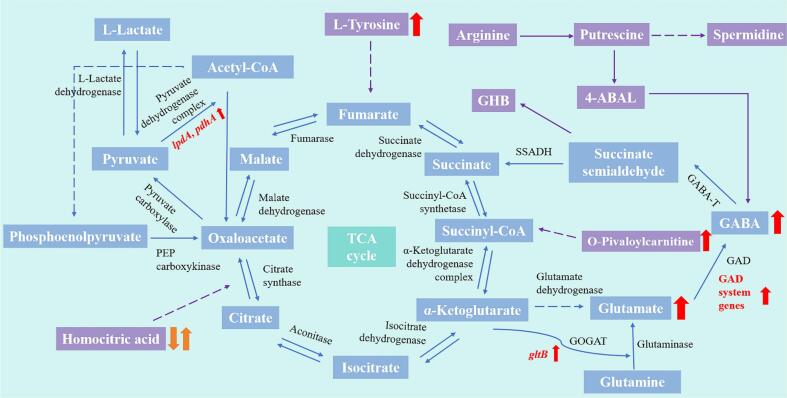
Proposed mechanism of GABA enhancement in *L. plantarum* JY7 under thermosonication. Coordinated upregulation of the GAD system, enhanced glutamate supply, and activation of energy metabolism drive increased GABA production.

To specifically address the transition from PLP to the acoustic‑thermal coupling field (US vs PLP), heatmap and correlation network analysis between these two groups were performed (Fig. 8 8e, f). In the comparison between the PLP and US groups, the PLP group exhibited significantly strengthened gene-metabolite associations related to specific lipid-signalling metabolism (e.g., prostaglandin precursors). The enrichment of secondary metabolites such as flavonoids provides direct evidence that cells activate endogenous antioxidant systems to cope with potential oxidative stress induced by the physical field [54]. Changes in lipid signalling molecules may be involved in regulating membrane fluidity, substance transport and cellular adaptability. In contrast, the US group exhibited upregulation of seven stress-responsive genes and accumulation of phosphoenolpyruvic acid, suggesting a distinct reconfiguration of glycolytic flux that supports sustained energy supply for GABA synthesis. These coordinated metabolic adjustments, including enhanced antioxidant capacity, membrane remodelling and energy reserve accumulation, represent the primary contribution of the acoustic-thermal coupling field. This field creates a protective and supportive cellular environment that maintains the high GABA production achieved during the PLP stage. Furthermore, the GABA yield in the US group was elevated beyond that of the PLP group, which demonstrates that the acoustic-thermal coupling field not only consolidated the metabolic state established by PLP but also exerted an incremental positive effect on GABA accumulation. The activation of these pathways aims to consolidate the high metabolic flux established during the PLP stage and, by enhancing long‑term cellular robustness and stability, ensure the sustainability of the high‑yield state. Focused analysis on the GABA synthesis pathway (Fig. 9) revealed that the genes encoding glutamate decarboxylase and its associated transporter protein were coordinately and significantly up-regulated, providing direct transcriptional evidence for the accumulation of GABA observed in metabolomics [64]. Concurrently, genes related to energy metabolism, amino acid transport, and stress response were also synchronously up-regulated. This indicates that the acoustic-thermal field activated a coordinated transcriptional regulatory network, which not only enhanced GABA synthesis capacity but also comprehensively strengthened cellular precursor supply, energy output and stress tolerance [1].

## 5 Conclusion

An acoustic-thermal coupling strategy was proposed and validated in this study to overcome the yield and efficiency bottlenecks in the traditional fermentation production of γ-aminobutyric acid (GABA) by lactic acid bacteria. *Lactiplantibacillus plantarum* JY7, isolated from fish intestine samples, was identified as a high-yield GABA-producing strain. Its GABA yield reached 1.0014 g/L after 60 h of fermentation in basal MRS medium supplemented with L-MSG. The strain also exhibited multiple probiotic properties, including acid tolerance, bile salt tolerance, and broad-spectrum antibacterial activity, highlighting its potential as a model strain for in-depth research. The optimal conditions were determined through the combined optimization of single-factor experiments and response surface methodology as 0.5 % L-MSG, 45 μmol/L PLP, 38℃ and an acoustic-thermal coupled field parameter of 150 W. Under these optimised conditions, the GABA yield reached 0.5104 mg/mL, which represented a 2.1-fold increase compared with the untreated control in pure MRS medium (0.243 mg/mL) under the same fermentation time (24 h). SEM observations revealed that the coupled treatment induced reversible damage and increased permeability in the cell wall and membrane structure. This provided direct morphological evidence for enhanced substrate uptake and product efflux. Integrated multi-omics analysis further revealed that the acoustic-thermal coupling field acts not only as an exogenous stimulus but also as a regulatory switch, triggering a series of progressively amplified metabolic reprogramming events. It is noteworthy that the acoustic‑thermal coupling field acts as an integrated physical stimulus. The observed transcriptional and metabolic reprogramming reflects a composite response to mechanical (cavitation, shear force) and mild thermal perturbations rather than a simple heat shock effect. At the transcriptional level, significant up-regulation of GAD system genes was observed, leading to a transient enhancement of glutamate decarboxylase transcriptional activity. With regard to energy and carbon skeleton metabolism, the pyruvate dehydrogenase complex, TCA cycle and purine synthesis pathways were co-activated, providing continuous ATP and reducing power support for the GABA decarboxylation reaction. These responses exhibited a clear temporal sequence, evolving from a nutritional foundation to cofactor-driven enhancement, and culminating in a physical field triggering metabolic network impact. Ultimately, metabolic flux was stably channeled towards the efficient GABA synthesis pathway. This strategy provides a reproducible paradigm for integrating physical field-assisted fermentation with systems biology. Genetic engineering remains the primary route for maximizing GABA titers, whilst thermosonication offers a complementary and rapid-deployment approach for enhancing native strain performance without genomic modification. Future work will focus on strain engineering (e.g., overexpression of *gadB/gadC*), combined with optimized thermosonication parameters to further bridge the yield gap with engineered systems.

## CRediT authorship contribution statement

**Wenying Yuan:** Writing – original draft, Methodology, Investigation, Formal analysis, Data curation. **Hao Zhang:** Supervision, Methodology. **Lixin Cai:** Writing – review & editing, Formal analysis. **Xiefei Li:** Visualization, Resources, Methodology. **Daodong Pan:** Methodology, Investigation. **Zhen Wu:** Methodology. **Jie Luo:** Writing – review & editing, Conceptualization. **Maiquan Li:** Formal analysis. **Xiankang Fan:** . **Hui Zhou:** .

## Declaration of competing interest

The authors declare that they have no known competing financial interests or personal relationships that could have appeared to influence the work reported in this paper.

## Appendix A Supplementary data

Supplementary Data 1 Additional tables including RT-qPCR primer sequences (Table S1), Box–Behnken design variables and experimental results (Tables S2–S4), and RNA-seq read mapping statistics (Table S5). The supplementary material has been re-uploaded accordingly.
