# Supplementary material for: Multi-omics analysis reveals the mechanism of enhanced GABA production in Lactiplantibacillus plantarum JY7 under acoustic and thermal coupling
Source: Ultrason Sonochem. 2026 Jul 21;132:107969. doi: 10.1016/j.ultsonch.2026.107969 (PMC13427605; doi:10.1016/j.ultsonch.2026.107969)
Supplement: Supplementary Data 1 — Additional tables including RT-qPCR primer sequences (Table S1), Box–Behnken design variables and experimental results (Tables S2–S4), and RNA-seq read mapping statistics (Table S5). The supplementary material has been re-uploaded accordingly. [file mmc1.docx]

Supplementary material

Table S1. Primer sequences for RT-qPCR

| Gene |  | Sequence |
| --- | --- | --- |
| 16S rRNA | F | 5’-CGTAGGTGGCAAGCGTTGTCC-3’ |
|  | R | 5’-CGCCTTCGCCACTGGTGTTC-3’ |
| *gadB* | F | 5’-TGGGARAAGTTCTGTRYCTACTGG-3’ |
|  | R | 5’-ACGTTCKYCARCCGGAAGTCCC-3’ |
| *gadC* | F | 5’-GCATTGTTCTACCAATGGATTCA-3’ |
|  | R | 5’-TGTGGTGCCGACCCTTCGGCACC-3’ |

Table S2. Levels and code of variables used in Box–Behnken design

| Variable | Coded levels | | |
| --- | --- | --- | --- |
|  | -1 | 0 | 1 |
| L-MSG (A, %) | 0.25 | 0.5 | 0.75 |
| PLP(B, μmol/L) | 25 | 50 | 75 |
| Ultrasonic power(C, W) | 100 | 150 | 200 |
| Ultrasonic temperature(D, ℃) | 32 | 37 | 42 |

Table S3. Box–Behnken experimental design and the results

| Test | A | B | C | D | GABA content (mg/mL) |
| --- | --- | --- | --- | --- | --- |
| 1 | -1 | -1 | 0 | 0 | 0.393417 |
| 2 | 1 | -1 | 0 | 0 | 0.403249 |
| 3 | -1 | 1 | 0 | 0 | 0.348654 |
| 4 | 1 | 1 | 0 | 0 | 0.423233 |
| 5 | 0 | 0 | -1 | -1 | 0.404051 |
| 6 | 0 | 0 | 1 | -1 | 0.418766 |
| 7 | 0 | 0 | -1 | 1 | 0.43719 |
| 8 | 0 | 0 | 1 | 1 | 0.441271 |
| 9 | -1 | 0 | 0 | -1 | 0.382232 |
| 10 | 1 | 0 | 0 | -1 | 0.358391 |
| 11 | -1 | 0 | 0 | 1 | 0.356213 |
| 12 | 1 | 0 | 0 | 1 | 0.436588 |
| 13 | 0 | -1 | -1 | 0 | 0.418395 |
| 14 | 0 | 1 | -1 | 0 | 0.43311 |
| 15 | 0 | -1 | 1 | 0 | 0.451686 |
| 16 | 0 | 1 | 1 | 0 | 0.408008 |
| 17 | -1 | 0 | -1 | 0 | 0.354466 |
| 18 | 1 | 0 | -1 | 0 | 0.409045 |
| 19 | -1 | 0 | 1 | 0 | 0.376229 |
| 20 | 1 | 0 | 1 | 0 | 0.404131 |
| 21 | 0 | -1 | 0 | -1 | 0.395148 |
| 22 | 0 | 1 | 0 | -1 | 0.429239 |
| 23 | 0 | -1 | 0 | 1 | 0.467689 |
| 24 | 0 | 1 | 0 | 1 | 0.401063 |
| 25 | 0 | 0 | 0 | 0 | 0.505447 |
| 26 | 0 | 0 | 0 | 0 | 0.511861 |
| 27 | 0 | 0 | 0 | 0 | 0.501985 |
| 28 | 0 | 0 | 0 | 0 | 0.498223 |
| 29 | 0 | 0 | 0 | 0 | 0.493788 |

Table S4. Analysis of variance for the quadratic response surface model

| Source | Sum of squares | Df | Mean square | F value | P value | Significance |
| --- | --- | --- | --- | --- | --- | --- |
| Modified model | 0.0613 | 14 | 0.0044 | 143.94 | < 0.0001 | Significant |
| A-L-MSG | 0.0042 | 1 | 0.0042 | 136.84 | < 0.0001 |  |
| B-PLP | 0.0006 | 1 | 0.0006 | 20.40 | 0.0005 |  |
| C-Ultrasonic power | 0.0002 | 1 | 0.0002 | 5.27 | 0.0377 |  |
| D-Ultrasonic temperature | 0.0019 | 1 | 0.0019 | 63.49 | < 0.0001 |  |
| AB | 0.0010 | 1 | 0.0010 | 34.47 | < 0.0001 |  |
| AC | 0.0002 | 1 | 0.0002 | 5.85 | 0.0298 |  |
| AD | 0.0027 | 1 | 0.0027 | 89.32 | < 0.0001 |  |
| BC | 0.0009 | 1 | 0.0009 | 28.04 | 0.0001 |  |
| BD | 0.0025 | 1 | 0.0025 | 83.42 | < 0.0001 |  |
| CD | 0.0000 | 1 | 0.0000 | 0.9300 | 0.3512 |  |
| A² | 0.0382 | 1 | 0.0382 | 1255.58 | < 0.0001 |  |
| B² | 0.0083 | 1 | 0.0083 | 273.92 | < 0.0001 |  |
| C² | 0.0093 | 1 | 0.0093 | 306.46 | < 0.0001 |  |
| D² | 0.0112 | 1 | 0.0112 | 366.79 | < 0.0001 |  |
| Residual | 0.0004 | 14 | 0.0000 |  |  |  |
| Lack of fit | 0.0002 | 10 | 0.0000 | 0.4937 | 0.8334 | Not significant |
| Pure error | 0.0002 | 4 | 0.0000 |  |  |  |
| Cor total | 0.0617 | 28 |  |  |  |  |
| Std. Dev. | 0.0055 | R² | 0.9931 | Adeq Precision | | 38.9714 |
| Mean | 0.4229 | R_Adj_² | 0.9862 |  | |  |
| C.V. % | 1.3039 | R_Pred_² | 0.9732 |  | |  |

Table S5. Summary of RNA-seq read mapping statistics for each sample against the *Lactiplantibacillus plantarum* reference genome

| Sample | Total clean reads (n) | Mapped reads (n) | Overall mapping rate (%) |
| --- | --- | --- | --- |
| Con1 | 7865616 | 6998101 | 88.97% |
| Con2 | 7810445 | 6957871 | 89.08% |
| Con3 | 7878756 | 7004269 | 88.90% |
| MSG1 | 7857863 | 7036739 | 89.55% |
| MSG2 | 7802060 | 7014615 | 89.91% |
| MSG3 | 7761543 | 6970049 | 89.80% |
| PLP1 | 7873287 | 7070106 | 89.80% |
| PLP2 | 7826444 | 7031088 | 89.84% |
| PLP3 | 7847893 | 7044543 | 89.76% |
| US1 | 7895564 | 7118081 | 90.15% |
| US2 | 7803576 | 7027858 | 90.06% |
| US3 | 7743229 | 7005917 | 90.48% |
